# CMR unveiling the cause of post CoVid-19 infection chest pain

**DOI:** 10.1007/s10554-021-02161-y

**Published:** 2021-01-28

**Authors:** Suzan Hatipoglu, Alexander R. Lyon, Dudley J. Pennell

**Affiliations:** 1 Royal Brompton Hospital, London, UK; 2 Imperial College London, London, UK

A 63 year old male presented with exercise induced chest pain 50 days after diagnosis of CoVid-19 infection (PCR positive) and was referred for cardiovascular magnetic resonance (CMR). Volumetric analysis showed high-normal indexed left ventricular volumes with low-normal ejection fraction of 60% (Supplementary Videos 1–4,* 3 long axis views and short axis cine stack*) and mild hypokinesia in the basal lateral wall. Native T1 values in the lateral wall were raised (1076 ms at 1.5 T, normal range 950–1050 ms, Panel A) and T2 values were borderline high (56 ms Panel B). Signal intensity was increased with a T2-short-tau-inversion-recovery sequence in the basal to mid lateral wall (Panels C and D, yellow arrows) with corresponding late gadolinium enhancement (Panels E and F, yellow arrow). Pericardial signal and thickness were normal with a small global pericardial effusion, but no CMR evidence of ventricular interdependence. There was no inducible myocardial ischaemia on perfusion imaging, with a normal global myocardial perfusion reserve of 2.8. Computed tomography (CT) pulmonary angiography was normal and there was non-obstructive atheroma on CT coronary angiography. Troponin and NT-pro BNP were normal, and d-dimer was elevated (930 ng/mL normal < 240).


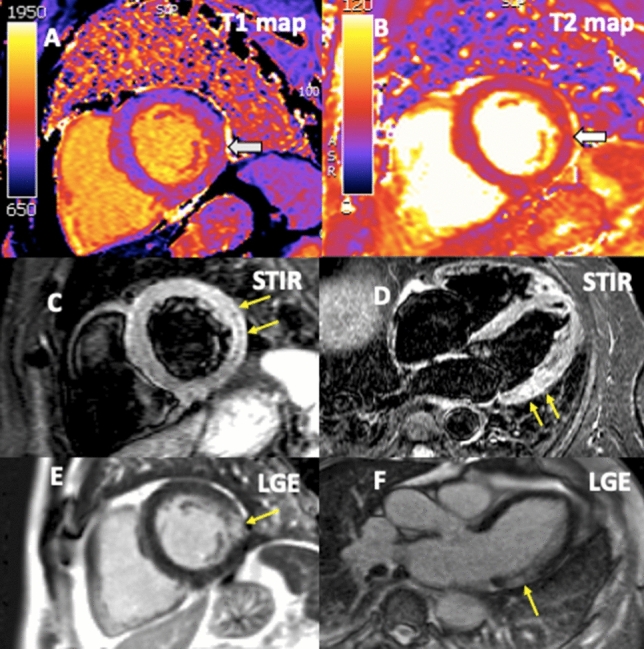


The CMR findings were diagnostic for myocardial oedema and acute/subacute myocarditis without ischaemia infarction. The myocarditis was presumed to be immune-mediated given the presentation after recovery from CoVid-19 and patient was empirically treated with oral steroids [1]. In repeat CMR after 2 weeks of treatment, tissue characterisation appearances were similar but wall motion abnormality in the basal lateral wall was no longer present and ejection fraction improved to 68%. This report highlights the clinical value of CMR to differentiate between possible causes of chest pain in CoVid-19 cases and demonstrates state-of-art symptom guided use in line with Society of Cardiovascular Magnetic Resonance recommendations [2].

## Supplementary Information

Below is the link to the electronic supplementary material.

Supplementary file1 (AVI 38576 KB)

Supplementary file2 (AVI 44087 KB)

Supplementary file3 (AVI 42476 KB)

Supplementary file4 (AVI 405762 KB)
